# *RABDAM*: quantifying specific radiation damage in individual protein crystal structures

**DOI:** 10.1107/S1600576718002509

**Published:** 2018-03-28

**Authors:** Kathryn L. Shelley, Thomas P. E. Dixon, Jonathan C. Brooks-Bartlett, Elspeth F. Garman

**Affiliations:** a Department of Biochemistry, University of Oxford, South Parks Road, Oxford OX1 3QU, UK

**Keywords:** radiation damage, atomic *B* factors, atomic displacement parameters, *B*_Damage_, *RABDAM*, Protein Data Bank, PDB, protein crystallography, computer programs

## Abstract

Radiation damage remains one of the major limitations to accurate structure determination in protein crystallography (PX). Despite the use of cryo-cooling techniques, it is highly probable that a number of the structures deposited in the Protein Data Bank (PDB) have suffered substantial radiation damage as a result of the high flux densities of third generation synchrotron X-ray sources. Whereas the effects of global damage upon diffraction pattern reflection intensities are readily detectable, traditionally the (earlier onset) site-specific structural changes induced by radiation damage have proven difficult to identify within individual PX structures. More recently, however, development of the *B*_Damage_ metric has helped to address this problem. *B*_Damage_ is a quantitative, per-atom metric identifies potential sites of specific damage by comparing the atomic *B*-factor values of atoms that occupy a similar local packing density environment in the structure. Building upon this past work, this article presents a program, *RABDAM*, to calculate the *B*_Damage_ metric for all selected atoms within any standard-format PDB or mmCIF file. *RABDAM* provides several useful outputs to assess the extent of damage suffered by an input PX structure. This free and open-source software will allow assessment and improvement of the quality of PX structures both previously and newly deposited in the PDB.

## Abstract 2

A program to measure the extent of specific radiation damage suffered by an individual protein crystal structure, suitable for running on any standard-format PDB or mmCIF file, is presented.

## 1. Introduction   

During X-ray diffraction data collection, the majority of the interacting X-rays are absorbed by the crystal under study, rather than being elastically scattered by it. These absorbed X-rays deposit energy within the sample, causing damage. The rate of accumulation of damage with dose (the energy absorbed per unit mass) can be reduced by cryo-cooling (Garman & Schneider, 1997 ▸), amongst other strategies. However, despite the use of such techniques, the high flux densities provided by third generation synchrotron sources have caused radiation damage to remain one of the major limitations to accurate structure determination in protein crystallography (PX), at both room and cryo-temperatures.

Radiation damage can be divided into two classes: global and specific. Global radiation damage occurs in reciprocal space, resulting in the degradation of the crystal lattice and thus a reduction in the quality of the diffraction data obtained from the crystal. Consequently, global radiation damage is detectable from changes in diffraction pattern reflection intensities; in particular it manifests as a gradual fading and an ultimate loss of high-resolution reflections.

Conversely, specific radiation damage occurs in real space, causing chemical changes as well as structural disordering within the asymmetric unit. At cryo-temperatures, these chemical changes have been observed to occur at conserved locations across a wide range of proteins in a relatively reproducible order that appears to correlate with a site’s electron affinity. With increasing dose, metallocentres are reduced; di­sulfide bonds are elongated and broken; glutamate and aspartate residues are de­carboxyl­ated; and the methyl­thio group is cleaved from me­thio­nine residues (Yano *et al.*, 2005 ▸; Burmeister, 2000 ▸; Ravelli & McSweeney, 2000 ▸; Weik *et al.*, 2000 ▸).

Specific radiation damage effects typically manifest at much lower doses than global damage effects; for example, previous studies have detected metal ion reduction and di­sulfide bond cleavage at doses as low as ∼0.35 MGy at 110 K (Corbett *et al.*, 2007 ▸) and ∼1 MGy at 100 K (Sutton *et al.*, 2013 ▸), respectively. These doses are well below the experimental dose limit of 30 MGy, which was measured as the average dose leading to a 30% loss in diffracting power of cryo-cooled apo- and holo-ferritin crystals (Owen *et al.*, 2006 ▸). In comparison, the dose absorbed by a crystal during a typical X-ray diffraction experiment at cryo-temperatures is usually of the order of several MGy (Garman, 2010 ▸). Therefore, many of the PX structures deposited in the Protein Data Bank (PDB; https://www.rcsb.org/) are likely to have suffered specific radiation damage.

If not identified and accounted for, the structural artefacts introduced by specific radiation damage can lead to incorrect biological conclusions being drawn from a PX structure. This issue is exacerbated by the fact that the residues most susceptible to specific radiation damage are commonly found at enzyme active sites. Furthermore, active site residues have often been found to be more prone to specific radiation damage compared with their non-active site counterparts (Weik *et al.*, 2000 ▸; Dubnovitsky *et al.*, 2005 ▸).

Consequently, there is a compelling need to accurately identify radiation damage artefacts within PX structures. Unfortunately, whereas global damage effects are readily detectable within the diffraction patterns collected from an individual PX structure, specific damage effects are typically much more difficult to recognize. Specific radiation damage is usually identified from differences between successive data sets collected from the same crystal, expressly as electron density loss (and/or gain) peaks within *F*_obs (high-dose structure)_ − *F*_obs (low-dose structure)_ difference maps. However, this method can only be applied to the small subset of cases where multiple data sets have been collected. This raises a problem therefore; specific radiation-damage-induced chemical artefacts are likely to be present within a substantial proportion of the X-ray diffraction structures deposited in the PDB, without an easy means of detection.

To address this issue, in earlier work we developed *B*_Damage_, a per-atom metric to identify and quantify potential sites of specific radiation damage within an individual PX structure (Gerstel *et al.*, 2015 ▸). The structural changes caused by specific radiation damage are accompanied by a loss of electron density, and thus (assuming atomic *B* factor, rather than occupancy, refinement) an increase in *B* factor, at the affected sites. However, there are multiple other variables that can affect the *B*-factor value of an atom, the most important of which is its mobility; the increase in atomic *B* factor caused by specific radiation damage is usually insufficiently large to separate these two effects.

There is a strong positive correlation between the mobility of an atom within a crystal structure and its packing density, *i.e.* the number of atoms within its local environment (Weiss, 2007 ▸). The *B*_Damage_ metric we previously defined is the full atomic isotropic *B* factor (herein referred to as *B* factor) corrected for the local packing density; the *B*_Damage_ value of an atom *j* is calculated as the ratio between its *B* factor and the average *B* factor of atoms 1 to *n* which occupy a similar packing density environment to that of atom *j*:



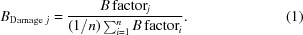

*B*_Damage_ is able to identify expected sites of specific radiation damage (as determined from difference map peaks from cases where multiple data sets have been collected) in several different PX structures (Fig. S1 in the supporting information). Therefore, the *B*_Damage_ metric has the potential to be highly useful in aiding crystallographers to assess the extent of specific radiation damage suffered by their PX structures. However, the code used to calculate *B*_Damage_ in our previous investigation was not made publically available, nor was it user-friendly.

Consequently, we have developed *RABDAM*, a program that enables the rapid calculation of *B*_Damage_ for all selected atoms within any standard-format PDB or mmCIF file. *RABDAM* has been designed to be highly flexible, and as such there are numerous user-specified options available to allow precise control of both the *B*_Damage_ calculation and the program output. However, default optimized parameter values are also provided to allow *B*_Damage_ to be calculated with minimal human intervention and expertise. Additionally, the organization of the *B*_Damage_ calculation around a dataframe data structure means that the program can be extended easily by developers.

In the following communication, we first describe the structure of the *RABDAM* code (v. 1.0). We then explain both how to run *RABDAM* and how to interpret the outputs obtained from the program. Finally, we discuss the wide range of potential applications of *RABDAM* and its limitations, plus expected future developments to the program.

## 2. The *RABDAM* code   

### 2.1. The *B*_Damage_ algorithm   

The *B*_Damage_ metric identifies potential sites of specific radiation damage as atoms with high *B*-factor values relative to other atoms that occupy a similar packing density environment in the context of the crystalline structure. The algorithm executed by *RABDAM* to calculate *B*_Damage_ (the ‘*B*_Damage_ algorithm’) can therefore be split into two stages: (i) calculation of packing density values, followed by (ii) calculation of the *B*_Damage_ metric itself.

There are multiple ways to calculate the packing density of an atom; here it is defined as its atomic contact number, *i.e.* the number of non-hydrogen atoms within a given radius, which in this case is set to 7 Å [as recommended by Weiss (2007 ▸) and in agreement with our own optimization experiments]. To calculate the packing density of each selected atom within an input PDB or mmCIF file of the asymmetric unit of a PX structure of interest, *RABDAM* generates a copy of the unit cell and translates it ±1 unit in all dimensions to build a 3 × 3 × 3 assembly of unit cells. Atoms in the 3 × 3 × 3 assembly that lie further than 7 Å from the asymmetric unit are discounted. The packing density of a selected atom *j* in the asymmetric unit is then calculated as the number of non-hydrogen atoms (incorporating atoms both selected for and excluded from the *B*_Damage_ calculation) within a 7 Å radius.

For the next step, *RABDAM* orders the selected asymmetric unit atoms by their packing density values. The *B*_Damage_ value of atom *j* is subsequently calculated as the ratio of its *B* factor to the average of the *B*-factor values of atoms classified, *via* a sliding window of size equal by default to 2% of the total number of atoms included in the *B*_Damage_ calculation, as occupying a similar packing density environment [see equation (1)].

**Figure 1:**
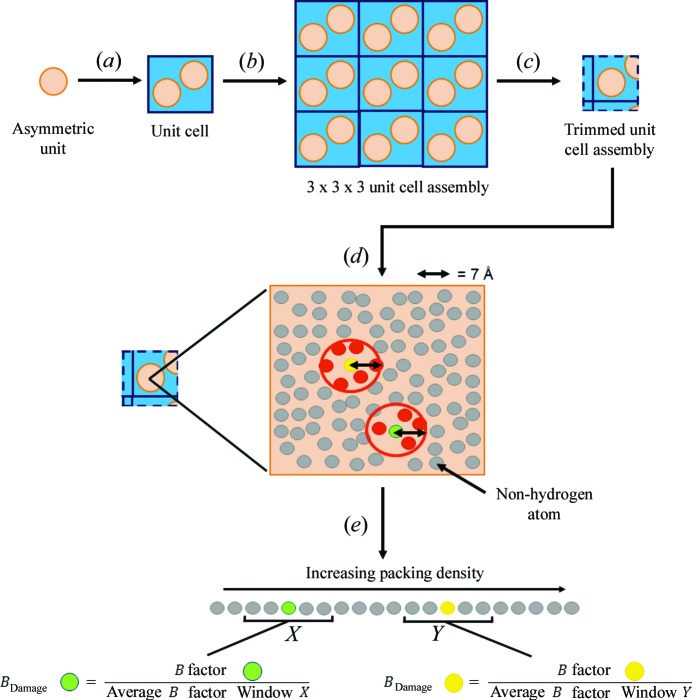
The *B*_Damage_ algorithm. Starting from the asymmetric unit coordinates of the PX structure of interest, *RABDAM* generates (*a*) a copy of the unit cell, followed by (*b*) a 3 × 3 × 3 assembly of unit cells. (*c*) Atoms in this assembly that lie further than 7 Å from the asymmetric unit are discarded. *RABDAM* then calculates (*d*) the packing density and subsequently (*e*) the *B*_Damage_ values of all user-specified asymmetric unit atoms. The unit cell and 3 × 3 × 3 unit cell assembly are represented here in two dimensions for clarity.

The *B*_Damage_ algorithm is summarized diagrammatically in Fig. 1 ▸.

### 2.2. Structure of the code   

**Figure 2:**
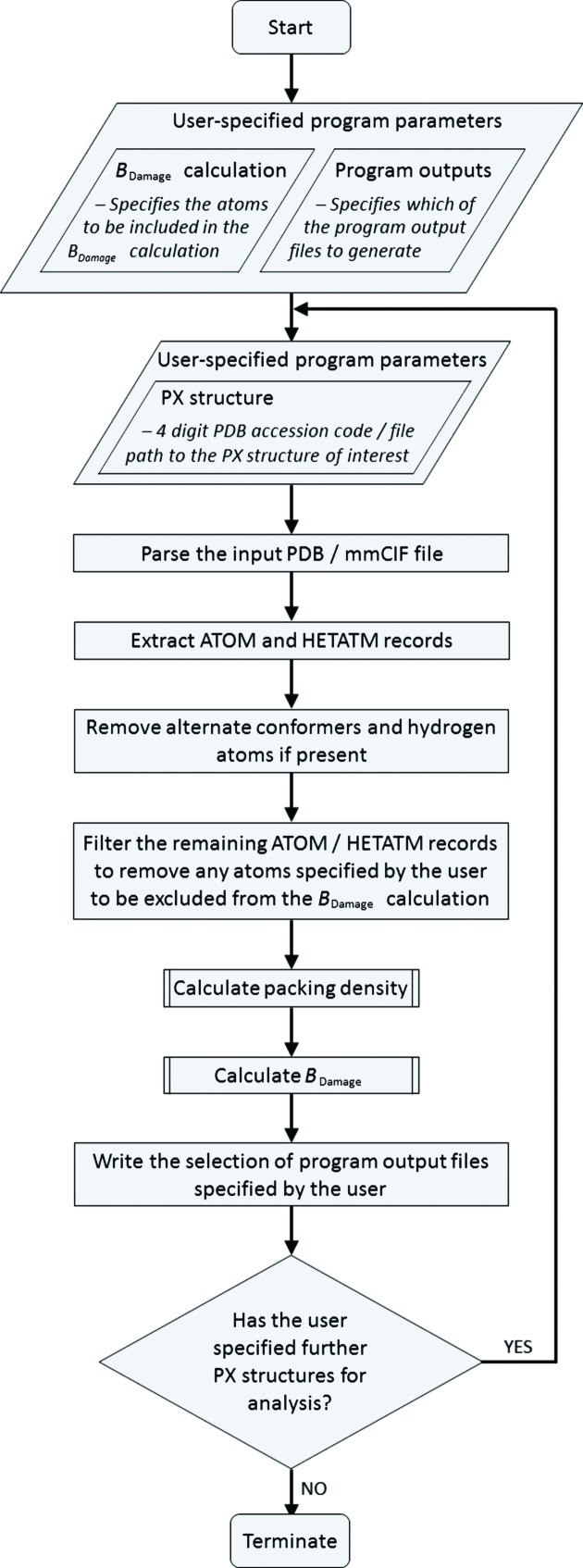
Flow chart illustrating the logical flow of the *RABDAM* code.

The logical flow of the *RABDAM* code, as shown in Fig. 2 ▸, starts by initializing the values of all user-specified program parameters. These variables include the identity of the input PX structure, as well as the subset of atoms to be incorporated in the *B*_Damage_ calculation (the ‘atoms of interest’) and the selection of program output files to be written (see §3). If the user specifies an input PDB accession code, *RABDAM* downloads the corresponding PDB and mmCIF files from the RCSB PDB web site; otherwise, if the user provides an input file path to a PDB/mmCIF file, *RABDAM* copies the file from the local machine.

After parsing the specified input PDB or mmCIF file (which in order to be processed correctly must conform to the standard PDB/mmCIF file formatting guidelines), *RABDAM* extracts the ATOM/HETATM records of the atoms of interest. As described in §2.1, *RABDAM* does not include hydrogen atoms in the *B*_Damage_ calculation. In addition, the program only considers a single conformer per amino acid residue (namely the highest occupancy conformer listed first in the input PDB/mmCIF file). This is because the occupancy values of alternate conformers are commonly not subject to refinement; hence comparisons between the *B*_Damage_ values of alternate conformers of the same residue would be potentially misleading given the correlation between occupancy and *B*-factor values. The exclusion of alternate conformers does not have a substantial effect upon the packing density values calculated for the retained atoms, since these values, when measured within a 7 Å radius, are typically in the range of 20 to 100 atoms.

*RABDAM* executes the *B*_Damage_ algorithm (see §2.1 and Fig. 1 ▸) to calculate the packing density and *B*_Damage_ values for the atoms of interest, which it writes to a dataframe data structure. The program then uses this dataframe to write the output files (see §4) selected by the user. *RABDAM* can be run in batch mode by specifying multiple PDB and/or mmCIF files for analysis.

### 2.3. Technical details of the code   

*RABDAM* is a command-line program written in the object-oriented programming language Python (the program is compatible with both Python 2.7 and Python 3.6). In the class-based object-oriented programming architecture, classes are employed to represent generalized concepts using a description of attributes and/or program code, whilst objects are instances of classes. This architecture is exploited in the *RABDAM* code to enhance its flexibility. As described in §2.2, *RABDAM* writes the results of the *B*_Damage_ calculation to a dataframe, which is then used to generate the output files specified by the user. The code is organized such that the dataframe is an object, thus allowing the user easy access to the raw data in a format that is readily manipulated. Furthermore, the organization of the output file calculations as class member functions, which take the dataframe as an argument, enables the user to easily incorporate their own functions into the program if they wish to generate alternative outputs. Owing to the precise definition of the *B*_Damage_ metric, it is not expected that this section of the code would be altered by users.

*RABDAM* is compatible with Linux, Macintosh and Windows operating systems. It takes approximately 1 min to perform the *B*_Damage_ calculation for a 200 kDa structure using a single processor (as estimated from tests performed on the Windows 7 operating system with a 3.70 GHz Intel i3-4170 processor). *RABDAM* currently has a dependence upon the *CCP4* suite program *PDBCUR* (Winn *et al.*, 2011 ▸), which it uses to generate the unit cell from the input PDB/mmCIF file of the asymmetric unit. The *RABDAM* program is available to download from GitHub (https://github.com/GarmanGroup/RABDAM), and has been incorporated in a recent update to the *CCP4* suite.

## 3. Program inputs   

**Figure 3:**
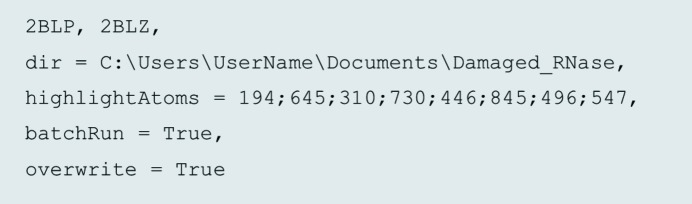
An example *RABDAM* input file. It directs *RABDAM* to perform *B*_Damage_ analysis for the PDB structures 2blp and 2blz [low- and high-dose structures, respectively, of ribonuclease A (Nanao *et al.*, 2005 ▸)], writing the output files to the user-specified directory ‘Damaged_RNase’, and highlighting atoms 194, 645, 310, 730, 446, 845, 496 and 547 (these are the sulfur atoms of the di­sulfide-forming cysteine residues) on the output *B*_Damage_ kernel density estimate produced for each structure. The input file also instructs *RABDAM* to skip to the next structure if it encounters any recognized program errors whilst analysing the current structure and to overwrite any previously existing files of the same name and located in the same directory as the output files written during the program run.

There are multiple program parameters that can be defined in the *RABDAM* input file (see Figs. 3 ▸ and S2 for examples) by the user, enabling straightforward and precise control over the selection of atoms incorporated in the *B*_Damage_ calculation, as well as over features of the program outputs. However, the only parameter which the user must define to initiate a *RABDAM* run is the identity/identities of one or more PX structures of interest. Notably, owing to the correlation between *B* factor and occupancy values, the only non-macromolecular atoms in the input structure(s) that have been subject to occupancy refinement should be those in alternate conformers (whose occupancy should sum to 1). Additionally, to enable damage detection, di­sulfide bonds should be refined as single occupancy rather than in alternate oxidized and reduced conformations. All other program parameters, if not specified by the user, default to pre-defined values, so that *RABDAM* can be run with minimal user intervention and expertise. Moreover, the default values are suitable for the vast majority of *RABDAM* runs.

As described in §1, *B*_Damage_ values are calculated from full atomic isotropic *B*-factor values, and it is these *B*-factor values that should be listed in the *B*-factor field of a structure’s ATOM/HETATM records according to both standard PDB and standard mmCIF file formatting guidelines. However, ∼10% of PDB/mmCIF files list alternative *B*-factor values in this field (Touw & Vriend, 2014 ▸); there are for instance many examples of PDB/mmCIF files of macromolecular structures refined with TLS groups that list residual instead of full isotropic *B*-factor values in this field.

The *B*-factor Databank (BDB) contains PDB files with full isotropic *B*-factor values in the ATOM/HETATM record *B*-factor field; all PDB entries with sufficient header information to determine the content of, and if necessary recalculate, the *B*-factor field are incorporated in the BDB (Touw & Vriend, 2014 ▸). *RABDAM* includes a regularly updated list of accession codes of structures deposited in the PDB with full isotropic *B* factors that has been downloaded from the BDB; the program flags a warning if the user specifies an accession code that is not on this list for *RABDAM* analysis.

Because calculation of the *B*_Damage_ metric involves making comparisons (of *B* factors) between atoms, it follows that *B*_Damage_ values are interrelated. This has important ramifications for the *B*_Damage_ calculation. One implication is that only structures for which data were collected to sufficiently high resolution to enable per-atom *B*-factor refinement are suitable for analysis with *RABDAM*.

Furthermore, inclusion/exclusion of atoms in/from the *B*_Damage_ calculation will affect the *B*_Damage_ values of all considered atoms, and hence great care must be taken in atom selection. As a result of the differences between the *B* factor to packing density ratios of protein, nucleic acid and heteroatoms, the *B*-factor values of these atom types are not suitable for comparison with one another. By default therefore *RABDAM* considers only the protein atoms listed within an input PDB/mmCIF file in its analysis. Although there are parameters facilitating finer control over atom selection, in most cases their use would be strongly discouraged given the interdependence of *B*_Damage_ values described above. However, an example of where these additional parameters might prove useful is in the exclusion of amino acid side chain atoms that have been modelled in the absence of electron density (and consequently have anomalously high *B*-factor values), such as can be found for instance in protein loop regions.

Descriptions of the roles of the most commonly used input parameters, how they are controlled and, if applicable, under what circumstances they should be altered from their default values are provided in Table 1 ▸, whilst an example input file is shown in Fig. 3 ▸. Full details of the input file parameters and grammar are provided in the program manual, which is available online at https://github.com/GarmanGroup/RABDAM.

## 4. Program outputs   

*RABDAM* will calculate *B*_Damage_ values for all selected atoms within the input PDB/mmCIF file; these data are displayed in various useful formats by the program output files listed in Table 2 ▸. The user is able to specify the particular subset of output files they would like *RABDAM* to generate *via* the command-line input to the program (a full description of which is provided in the online program manual).

Experimentally, *B* factors do not scale between different structures *via* any consistently observed relationship with either disorder or damage. This means that there can be variation between the (normalized) *B*-factor, and correspondingly the *B*_Damage_, distributions of equally damaged PX structures. Consequently, it is not possible to specify a universal threshold value of *B*_Damage_ above which an atom should be considered to have suffered specific radiation damage, and furthermore *B*_Damage_ values cannot be fairly compared between different structures. In light of these limitations, *RABDAM* also calculates the *B*_net_ metric, a derivative of *B*_Damage_ that summarizes the extent of specific radiation damage suffered by the input PX structure; *B*_net_ values are proportional to dose and hence, unlike *B*_Damage_ values, are comparable between proteins. Validation of the *B*_net_ metric will be provided in a future publication.

Moreover, because variables other than mobility, for example fluctuations in the quality of the electron density data throughout the structure, can have an impact upon the *B*-factor value of an atom, there is not a perfect (positive) correlation between specific radiation damage and *B*_Damage_. Atoms with higher *B*_Damage_ values are therefore more likely to be, but are not necessarily, damaged. Accordingly, the *B*_Damage_ metric is not expected to be used to definitively categorize atoms as ‘damaged’ or ‘undamaged’. Instead, it is intended that crystallographers should use this metric, in conjunction with prior knowledge of the atom types that are susceptible to the chemical changes caused by specific radiation damage (see §1), to identify atoms within their structures that are more likely to be damaged. This information can then be used to assist both modelling and biological interpretation of the structure (see §5).

## 5. An example *RABDAM* run   

**Figure 4:**
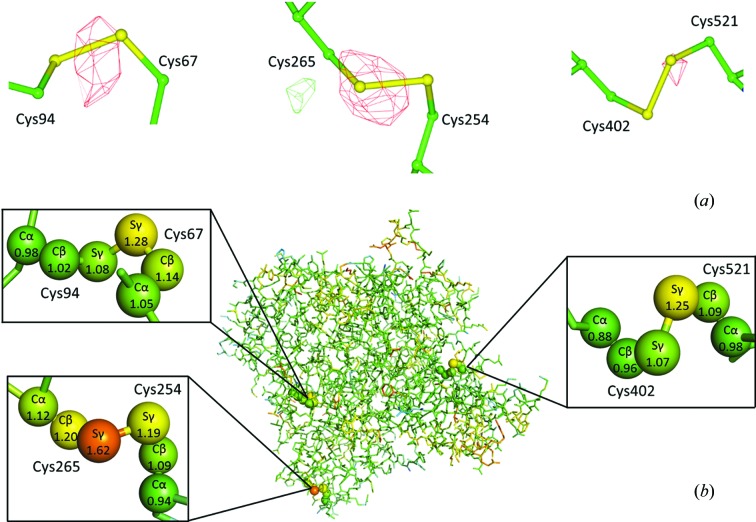
The sites of specific radiation damage present in the first two data sets of the radiation damage series collected from a crystal of acetyl­cholinesterase by Weik *et al.* (2000 ▸) are detected by *RABDAM*. (*a*) The *F*_obs (data set 1)_ − *F*_calc (data set 1)_ difference map, contoured at ±3σ in green/red. (*b*) The acetyl­cholinesterase structure coloured by *B*_Damage_ from blue (= low) through green to red (= high), also highlighting the individual *B*_Damage_ values of the cystine side chain atoms. The pattern of damage to the three intrachain di­sulfide bonds of acetyl­cholinesterase detected in (*a*) correlates with the *B*_Damage_ values calculated in (*b*). All molecular graphics images were rendered using *CCP4mg* (McNicholas *et al.*, 2011 ▸).

The results obtained from running *RABDAM* upon the first two data sets (PDB accession codes 1qid and 1qie) in a radiation damage series collected from a crystal of *Torpedo californica* acetyl­cholinesterase (Weik *et al.*, 2000 ▸) are presented in Fig. 4 ▸. These two data sets were calculated to have absorbed doses of ∼10 MGy (1qid) and ∼20 MGy (1qie). The *RABDAM* input file for this analysis is provided in Fig. S2. Note that, because *B* factors were refined per residue in the original work (whereas the *B*_Damage_ calculation requires per-atom *B*-factor refinement, see §3), *RABDAM* was run on the updated, per-atom re-refined and rebuilt structures downloaded from the *PDB_REDO* server (Joosten *et al.*, 2014 ▸) as opposed to the original structures deposited in the PDB.

There is very good agreement between the results generated by *RABDAM* as compared with those presented in the original publication. Weik *et al.* observed, from analysis of *F*_obs_ − *F*_calc_ difference maps, that the Cys254–Cys265 bond was the most readily damaged of the three intrachain di­sulfide bonds in acetyl­cholinesterase. Damage was already detectable within the first data set, with an especially prevalent density loss peak centred upon the Cys256 sulfur atom (Fig. 4 ▸ *a*). Correspondingly, the *B*_Damage_ value of the Cys256 sulfur atom is found to be substantially higher than those of the other cysteine sulfur atoms (plus the vast majority of other atoms) in the first data set (Fig. 4 ▸ *b*).

Moreover, Weik *et al.* observed a large increase in the *B*-factor values of Cys, Asp and Glu side chains in comparison with other residue types. In particular, they noted an ∼30% increase in the *B* factors of the side chains of both the surface residue Glu306 and the active site residue Glu327 between the first two data sets. Furthermore, there are large electron density loss peaks around the carboxyl group atoms of these residues present in the *F*_obs (data set 2)_ − *F*_obs (data set 1)_ difference density map (although similar peaks are not detectable within the *F*_obs_ − *F*_calc_ maps of the individual data sets). Accordingly, the *B*_Damage_ values (both raw and rank) of the carboxyl group atoms of each of these residues are found to increase substantially between the first and second data sets. These results are shown in Table 3 ▸.

Therefore, this analysis clearly demonstrates how *B*_Damage_ can identify sites of specific radiation damage that are otherwise undetectable within an individual data set, thus aiding the biological interpretation of a PX structure.

## 6. Discussion   

An inability to detect the majority of the structural and chemical changes induced by specific radiation damage within individual PX structures has probably resulted in a number of the structures deposited in the PDB containing unnoticed radiation damage artefacts. To address this issue, we have developed *RABDAM*, a flexible and user-friendly program that calculates our previously defined *B*_Damage_ metric to assess the damage suffered by all selected atoms within any standard-format PDB/mmCIF file.

*RABDAM* has multiple prospective applications in the field of protein crystallography. Its primary application, as demonstrated in §5, is in enabling the identification of potential localized sites, as well as the total extent, of specific radiation damage within individual PX structures both newly and previously deposited in the PDB. This analysis will be highly valuable in informing the biological conclusions that are drawn from a PX structure.

Furthermore, owing to its short run time and widely applicable default parameter values, *RABDAM* also has potential applications in the large-scale statistical analysis of specific radiation damage within the PDB. For instance, in earlier work we used *B*_Damage_ to analyse the damage present within a data set of 2704 PDB structures. From this investigation we identified differential radiation damage sensitivity of di­sulfide bond types (spiral, hook and staple), as well as a (positive) correlation between specific damage susceptibility and solvent accessibility (Gerstel *et al.*, 2015 ▸).

Identifying variables that affect specific damage propensity has proven difficult within previous radiation damage series studies owing to the small sample sizes involved. Consequently, there remains much controversy in the field as to whether certain variables, such as p*K*_a_ and solvent accessibility, in general affect the susceptibility of a site to specific radiation damage or not (Holton, 2009 ▸). *RABDAM* has the capacity to open up the entire PDB for statistical analysis of specific radiation damage, and so provide answers to these questions.

Such an approach would complement previous radiation damage series studies of individual model PX structures. Moreover, it would focus directly upon the radiation damage suffered by an archetypal PDB structure, thus avoiding the issue of extrapolation of results back from uncharacteristically high dose data sets that is faced by many radiation damage series studies. However, it could encounter difficulties resulting from the lack of available dose values for the vast majority of PDB structures, as well as from the substantial variation that is present within any large data set selected from the PDB; consequently, this approach is likely to supplement, rather than supersede, radiation damage series studies.

Similarly, the *B*_Damage_ metric complements, instead of superseding, *F*_obs (high dose)_ − *F*_obs (low dose)_ difference density analysis for the small subset of cases in which multiple data sets have been collected. As shown in §5, the two methods give similar results as expected, but they are not identical. This reflects the fact that *B*-factor values are able to capture structural disorder that difference electron density analysis cannot; some of this is likely to be noise, but some may be damage induced. However this also means that *B*_Damage_ is not as sensitive as difference density analysis to the well established damage-induced chemical changes. Therefore, a combination of *B*_Damage_ and difference density analysis is likely to identify damage within multiple data sets more effectively than either technique in isolation.

As described in §3, because *B*_Damage_ is defined as a per-atom metric, it can only be calculated for structures of sufficiently high resolution to be subject to per-atom *B*-factor refinement. Additionally, the interrelationship between resolution and *B*-factor values means that the *B*_Damage_ metric is not equally sensitive to damage across the resolution range over which it can be calculated; this reflects the fact that, as structure resolution decreases, the interdependence of the *B*-factor values of neighbouring atoms increases (Schneider *et al.*, 2014 ▸). Similarly, the radius and strength of the restraints applied during *B*-factor refinement will also affect the extent of the correlation between the *B* factors of adjacent atoms. Consequently, although theoretically the isotropic *B*-factor value of an atom *j* is directly related to its mean square isotropic displacement *u*,
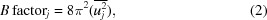
experimental differences between the resolution and/or refinement protocols of PX structures prevent meaningful comparison of their *B*-factor, and accordingly also their *B*_Damage_, values.

The sensitivity of *B*-factor, and hence *B*_Damage_, values to specific radiation damage is also affected by structure refinement quality; poor agreement between the experimental data and the model fitted to it will increase the level of noise in the *B*-factor values associated with the model. Nevertheless, alternative methods of radiation damage detection would be similarly affected by such error, and moreover only very poorly refined structures will contain adequate levels of noise to prevent detection of radiation damage. However, for any large-scale analysis of PX structures using *RABDAM*, we would recommend either downloading structures from the *PDB_REDO* server, or alternatively subjecting structures downloaded from the PDB to an initial round of *B*-factor refinement, in order to reduce the likelihood of refinement errors having an impact on the analysis.

Future planned developments to the program include its extension to cover nucleic acids. At present *RABDAM* is only able to assess the specific radiation damage suffered by the protein component of a macromolecular crystal structure; however, like proteins, nucleic acids suffer damage at conserved sites in a predetermined order. We will also explore options for enhancing the measurement of packing density to consider larger-scale motions within the asymmetric unit, for example by measuring the atomic contact number over a range of different radii. Additionally, we intend to use *RABDAM* to analyse the increasing number of room-temperature PX structures deposited in the PDB. Such analysis would help to address the shortfall of room-temperature radiation damage series, thus allowing a comprehensive comparison with the patterns of radiation damage susceptibility previously established at cryo-temperatures.

## 7. Summary   

*RABDAM* is a free and open-source program that facilitates the straightforward and rapid detection of specific radiation damage within individual PX structures, a task that has previously proven extremely difficult. Use of this software will enable protein crystallographers to readily assess the extent of specific radiation damage suffered by their structures, thus helping to improve both the quality of the PX structures newly deposited in the PDB and the biological conclusions that are drawn from these structures in the literature. In addition, the results output by *RABDAM* will help to inform assessments of the quality of previously deposited structures subject to atomic *B*-factor refinement.

There are numerous programs dedicated to the multiple stages of crystal structure solution. In contrast, there are very few programs concerned with the analysis of radiation damage in crystallography, and as far as we are aware *RABDAM* is the first program to enable the identification and quantification of specific radiation damage within individual PX structures. Therefore, it is hoped that the availability and ease of use of *RABDAM* will help to increase awareness within the protein crystallography community of the likelihood of specific radiation damage artefacts within their structures.

## Supplementary Material

Supporting Figures S1 and S2. DOI: 10.1107/S1600576718002509/ap5024sup1.pdf

## Figures and Tables

**Table 1:** The parameters most commonly required when running *RABDAM* The program parameters whose names are in quotation marks are set using that name in the *RABDAM* input file. A full list of program parameters plus a description of their functions is provided in the online program manual.

Program parameter	Description
Input PX structure(s)	Defines the PX structure(s) to be analysed, *via* either its four digit PDB accession code or a local file path. This parameter must be defined to initiate a *RABDAM* run (no default).
‘dir’	Defines the directory to which program output files are written. If not specified, it defaults to the current working directory.
‘highlightAtoms’	Defines which atoms to highlight on the output kernel density estimate of the *B*_Damage_ distribution (default = none).
‘batchTermination’	Defines whether the program should terminate (default) or skip to the next structure when it encounters a recognized program error during a run.
‘overwrite’	Allows the user to instruct *RABDAM* to overwrite all previously generated output files of the same name and located in the same directory as those to be written in the current program run (by default user input is required for each case encountered).

**Table 2:** The output files generated by *RABDAM* All six files are written by default. However, it is possible for the user to select a subset *via* the command-line input to the program (described in the online program manual).

Output file(s) abbreviation	Description
kde	A kernel density estimate (Rosenblatt, 1956 ▸; Parzen, 1962 ▸) of the distribution of *B*_Damage_ values calculated for the input PX structure.
bdam	A PDB file in which the *B*-factor column of the ATOM/HETATM records is replaced by ln(*B*_Damage_) values (thus allowing the structure to be uniformly coloured by *B*_Damage_ using molecular graphics software such as *PyMol*; https://pymol.org/), plus an mmCIF file in which a *B*_Damage_ column has been appended to the ATOM/HETATM records.
csv	A csv file listing the properties (both those in the input PDB/mmCIF file and those calculated by *RABDAM*) of all selected atoms in the input PX structure.
bnet	The value of the *B*_net_ metric, plus a kernel density estimate of the atoms from whose *B*_Damage_ values it was calculated.
summary	An html file summarizing the results displayed in the other four output files.

**Table 3:** Illustration of the good agreement between *B*_Damage_ and other measures of specific radiation damage in an example damaged data set The damage to the Glu306 and Glu327 carboxyl groups that occurred between the first and second data sets collected from an acetyl­cholinesterase crystal by Weik *et al.* (2000 ▸) can be identified from the large increase in the *B*-factor values of these atoms between the two data sets, and from the large electron density loss peaks (measured using the *D*_loss_ metric; Bury *et al.*, 2016 ▸) present at these sites in the *F*_obs (data set 2)_ − *F*_obs (data set 1)_ difference map. This damage can also be detected within data set 2 from the high *B*_Damage_ values of the affected atoms. Each number in the table is equal to the mean average of the values of the relevant damage measure, either for the O∊ atoms of the pertinent glutamate residue, or for all atoms, as appropriate. The total number of atoms analysed in the acetyl­cholinesterase structure was 4244. Non-bracketed and bracketed numbers refer to raw and rank values, respectively. All raw values are provided to three significant figures, whilst rank values are exact.

	*B* factor (Å^2^)			*B*_Damage_	
Atoms	Data set 1	Data set 2	*D*_loss_ (e Å^−3^)	Data set 1	Data set 2
Glu306	32.6 (316.5)	41.7 (146)	2.08 (44.5)	1.15 (569.5)	1.37 (173.5)
Glu327	23.5 (1207.5)	32.2 (404)	1.81 (57.5)	1.18 (438)	1.53 (76.5)
All atoms	22.8	24.0	0.109	1.00	1.00
