# Supplementary material for: RABDAM: quantifying specific radiation damage in individual protein crystal structures
Source: J Appl Crystallogr. 2018 Mar 28;51(Pt 2):552–9. doi: 10.1107/S1600576718002509 (PMC5884390; doi:10.1107/S1600576718002509)
Supplement: Supplementary file 1 [file j-51-00552-sup1.pdf]

RABDAM: quantifying specific radiation damage in individual protein crystal structures

Kathryn L. Shelley*, Thomas P. E. Dixon, Jonathan C. Brooks-Bartlett and Elspeth F. Garman*

Department of Biochemistry, University of Oxford, South Parks Road, Oxford, OX1 3QU, United Kingdom.

* Correspondence e-mails: kathryn.l.shelley@gmail.com, elspeth.garman@bioch.ox.ac.uk

Supporting information

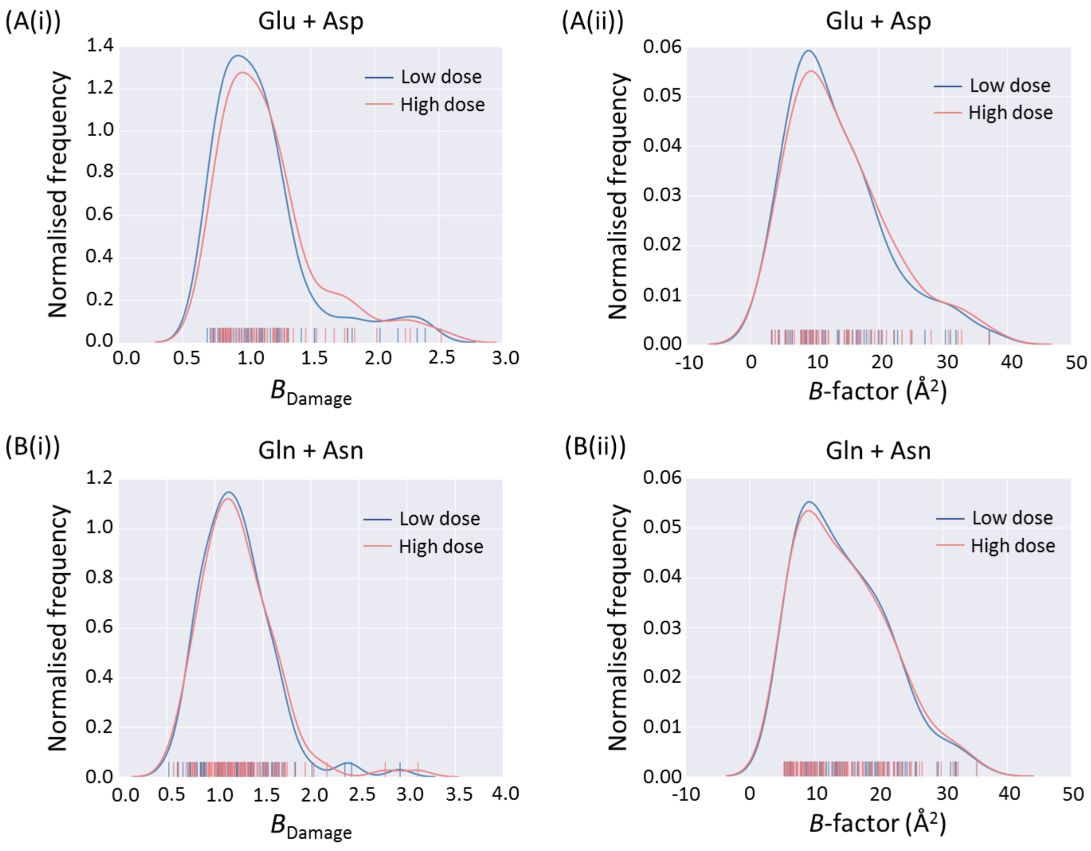

Figure S1: B_{Damage} highlights expected sites of specific radiation damage, which are not readily detectable from *B*-factor alone, in PX structures. **(A)** There is a larger difference between the **(i)** B_{Damage} as compared to the **(ii)** *B*-factor distributions of Asp Oδ1 and Glu Oε1 atoms for high- (pink) as compared to low- (blue) dose datasets. **(B)** In contrast, there is little discernible difference between both the **(i)** B_{Damage} and **(ii)** *B*-factor distributions of Asn Oδ1 and Gln Oε1 atoms (which are not expected to be sites of specific radiation damage induced chemical changes). The low- and high-dose distributions relate respectively to the low- and high-dose datasets collected from each of the six proteins (elastase, ribonuclease A, thaumatin, trypsin, lysozyme and insulin) studied in Nanao *et al.* (2005). Plots are smoothed via a kernel density estimator; B_{Damage} values of individual atoms are represented as rug plots, and were calculated using default program parameter values (*i.e.* a packing density radius of 7 Å and a sliding window size of 2% – see section §2.1 and the online program manual).

```
C:\Users\UserName\Documents\RABDAM_test_output\1QID_PDB_REDO.pdb,
C:\Users\UserName\Documents\RABDAM_test_output\1QIE_PDB_REDO.pdb,
dir = C:\Users\UserName\Documents\RABDAM_test_output,
highlightAtoms = 2376-2377;2538-2539
```

Figure S2: The RABDAM input file for the B_{Damage} analysis, presented in Figure 4 and Table 3, of the first two datasets in the *Torpedo californica* acetylcholinesterase radiation damage series collected by Weik *et al.* (2000). Because the original structures from this series deposited in the PDB were not subjected to per-atom *B*-factor refinement, as is a requirement for B_{Damage} calculation, the analysis was instead performed upon the updated, per-atom refined structures downloaded from the PDB_REDO databank; hence the input structures are specified by local PDB file paths as opposed to PDB accession codes. The highlightAtoms parameter is used to highlight the B_{Damage} values of the Oε atoms of Glu36 and Glu327 on the output kernel density estimate.
